# Nerve-Blocking as a Substitute for General Anesthesia in Surgical Operations

**Published:** 1913-12-15

**Authors:** M. L. Harris

**Affiliations:** Chicago


### NERVE-BLOCKING AS A SUBSTITUTE FOR GENERAL ANESTHESIA IN SURGICAL OPERATIONS.

M. L. HARRIS, M.D., CHICAGO.

Notwithstanding the inestimable advantages and blessings of general anesthesia as a method of inducing insensibility for surgical purposes, it is not without its serious drawbacks. In addition to the fact that most persons shrink from the idea of losing consciousness, general anesthesia has attached to it not only unpleasant after-effects, such as nausea, vomiting, headache, pulmonary irritation, pneumonia, suppression of urine, etc., but also the occasional, unavoidable sudden death.

It has long been the hope of surgeons and experimenters that some method of inducing insensibility to pain might be discovered which would be free from all the objectionable features of practically all the present known methods of inducing general anesthesia, and this hope seems now to be realized. Physical pain is the perception of afferent impulses received from that portion of the body which is the subject of injury. If no impulses reach the brain from the injured part, no pain is felt. The impulses or stimuli are transmitted from the periphery to the centers exclusively by the sensory nerves. If the power of the nerves to transmit these impulses be temporarily suspended by any means, any amount of injury may be inflicted to the parts supplied by the nerves whose function is suspended without the patient being conscious of such injury. The newer method of inducing anesthesia consists in suspending temporarily the power of the nerves to transmit afferent impulses and is called nerve-blocking. Nerve-blocking is produced by infiltrating a small portion of the nerve somewhere in its course between the periphery and the spinal cord with some substance which temporarily suspends its action. Not only does nerve-blocking prevent absolutely all pain from the region blocked, but it likewise prevents completely all general shock from any injury received to the parts blocked.

Shock may be defined as a condition in which there has been a sudden or rapid discharge of the potential energy of the nerve-centers, the result of afferent impulses or stimuli, due to peripheral injury. Without afferent stimuli, shock cannot be induced. By blocking the nerve to the passage of afferent stimuli, shock as an element in peripheral injury is entirely and completely eliminated. A leg for instance, the nerves from which have been blocked, may be crushed without the slightest evidence of pain or shock to the patient. As a surgical operation in a sense is simply an injury to some part of the body, it necessarily follows from what has preceded that a surgical operation performed on any portion of the body the nerves from which have been blocked will be unaccompanied by either pain or shock. During general anesthesia no pain is perceived, but shock is always induced, the anesthetic not withstanding. Every surgeon knows that shock is always present to a greater or less degree, in all operations performed under general anesthesia, the degree of shock depending on the amount of injury done and the duration of the operation, but it remained for Crile to demonstrate by experiment that even under profound general anesthesia afferent impulses are still received by the nerve-centers and that the potential energy of these centers is discharged by those impulses and thus shock results. Of course the shock is by no means so great as without the anesthetic, but it is an unavoidable element in every operation performed under general anesthesia, while under complete nerve-blocking shock is absolutely and entirely eliminated. It may seem almost incredible to many that the flesh can be cut and the bone sawed with the patient in full consciousness, yet perceiving no pain and suffering no shock; but such is a fact. That consciousness is retained in these operations may seem to many to be an objection to the method, but I think that in the majority of the cases it is a distinct advantage. There is no unpleasant psychic effect, as might be supposed, and even in nervous persons and children I have found no trouble in this respect. In addition to the fear of loss of consciousness the horror which many persons have of an operation is based on the fear of pain. If unconsciouness and all pain and shock be eliminated the operation has been robbed completely of all its terrors; and this is exactly what nerve blocking does.

Some months ago I began doing operations under nerve-blocking. The method proved so satisfactory in every way that its use was rapidly extended, until at present I do almost all of my operations by this method. and the use of a general anesthetic has become a rare exception. During this time I have operated on carcinomas of the face; tubercular glands of the neck; goiters and other tumors of the neck; carbuncles of the back of the neck; numerous affections of the extremities, including the plating of fractures; tumors of the abdominal wall; hernias; appendectomies, both acute and interval; gastrostomies; gastro-enterostomies; acute intestinal obstruction, due to volvulus and peritoneal bands; ovarian tumors; uterine fibroids; suprapubic and perineal prostatectomies; movable kidney; stones in the ureter and bladder; affections of the rectum, perineum and vagina, etc. It is unnecessary to give in detail all of these operations, but a few may be mentioned briefly to show what can be accomplished and how little effect operations done under nerve-blocking have on the patient.

### REPORT OF CASES.

CASE 1. Mrs. R., aged 42, had, I think, the largest goiter that I ever attempted to remove. It was composed of three distinct masses, that on the right side extending so high as to displace to lobe of the ear; that in the midline extending from the chin to the sternum, while that on the left side extended consideralby below the upper end of the sternum. It compressed the trachea so that she had great trouble in breathing. The operation was a difficult one and took nearly an hour and a half to complete. The patient talked to me repeatedly during the operation and expressed herself as suffering no pain. She left the table with a pulse of 88; it was usually 80 during the several days she was in the hospital before the operation. At the end of the operation she asked for a drink of water, which was given her. The next day she requested to be allowed to sit up, and made a rapid recovery.

Case 2.—Miss G., aged 12, had been suffering for some time with a large bunch of tuberculous lymph-nodes of the neck which had broken down and had been partially operated on by some one before. The dissection was a difficult one and required the removal of a portion of the jugular vein. It was done by blocking the cervical plexus and not a particle of a general anesthetic was given. Twice during the operation she was asked if she would like to be put to sleep, but she said, "No, go ahead and finish the operation." It is very evident that she suffered no pain, for being but a child of 12 she could not have controlled herself had she had any great pain. She had had ether for the first operation, which was small compared to this one, but she said she would not think of taking ether again for an operation.

Case 3.—Mr. K., aged 56, had marked obstruction of the pylorus; fully 50 per cent. of a barium meal was still found in the stomach after twenty-four hours as shown by the Roentgen ray. He vomited daily and had lost many pounds in weight. A diagnosis of carcinoma of the stomach was made. The abdomen was opened under nerve-blocking. No mass was found in the stomach or the pylorus, and there were no glandular metastases. The stomach was opened and explored thoroughly. The stenosis of the pylorus which was very marked was evidently benign in its origin. The stomach was closed and the usual posterior gastroenterostomy done. When the patient went to the operating room his pulse was 72, and on his return from the operating-room it was 74. He expressed himself as scarcely knowing that he had been operated on.

Case 4.—Mrs. M., had a large multilocular ovarian cyst, the size of an eight months' pregnancy. Operation performed under nerve-blocking. Extensive adhesions were found, fixing the tumor to the left parietal wall. The patient conversed during the operation and the only thing she said she felt was a peculiar drawing sensation in the abdomen when the tumor was turned out. She left the operating-room smiling and talking pleasantly with the nurse. She wanted to get up the next day.

Case 5.—Mr. G., had a small sharp-angled stone lodged in the lower end of the left ureter, which was giving him a great deal of trouble. The nerves supplying the left lower portion of the trunk and pelvis were blocked. The usual extraperitoneal incision for exposing the lower ureter was made. The ureter was followed down as far as possible, but the stone could not be felt. The peritoneum was opened and the ureter palpated down to the bladder, but the stone still escaped detection. The peritonuem was closed and the incision carried downward and inward until the fundus of the bladder could be reached. The ureter was opened at the brim of the pelvis and a long probe introduced. The bladder was opened so that the finger could be inserted, and the stone could now be felt between the end of the probe in the ureter and the finger in the bladder. After some manipulation the stone was finally pushed on into the bladder and removed. The operation was long and tedious. The patient's pulse at the end of the operation was 86, during the several days that he was in the hospital before the operation his pulse had ranged from 76 to 80.

These cases are sufficient to show what can be done under nerve-blocking and to show the entire absence of shock even in very severe and prolonged operations. This absence of shock is very remarkable indeed. Not a single patient on whom I have operated under nerve-blocking has expressed a regret at not having taken a general anesthetic, but all have been delighted with the result and every one has stated that were he obliged to submit to another operation, he would not think of taking a general anesthetic.

The technic of the method is quite simple. The nerves supplying the part are infiltrated for a short distance somewhere between the part to be operated on and the spinal cord. The method is entirely different from the old intraspinal anesthesia. There are several substances which will block nerves in this way, among which may be mentioned cocain, the various forms of eucain, stovain, novocain, quinin and urea hydrochlorid, etc. Some of these are so toxic as to be unsuited to the purpose. The substances which I have used are novocain and quinin and urea hydrochlorid, the former in solution 0.0025; 0.01 with the latter 0.0025; 0.005 solution. These substances are but very slightly toxic and may be used to the extent of several hundred cubic centimeters, depending on the strength of the solution. The exact points of injection naturally depend on the region to be blocked. The idea is to block all of the nerves supplying the part to be operated on. One must have an accurate knowledge of anatomy and some experience in order to reach the right points successfully. After the injection, it requires from five to thirty minutes for the anesthesia to become complete, depending on the place of injection and the size of the nerve to be infiltrated. As the method requires some skill and practice to secure perfect results, we see opened up here a new field for the expert anesthetist or nerve-blocker, one who in a busy clinic can have the patient ready for the operator at the right time. The method is so simple, so successful, and possesses so many advantages, that it opens up a new era in surgery and I believe marks the passing of the general anesthetic in surgical operations. Fuller details regarding technic and points of injection will be enlarged on in a future paper.—*Journal A. M. A.*, September 27, 1913.

